# Pediatric Obesity and Eating Disorders Symptoms: The Role of the Multidisciplinary Treatment. A Systematic Review

**DOI:** 10.3389/fped.2019.00123

**Published:** 2019-04-03

**Authors:** Rachele De Giuseppe, Ilaria Di Napoli, Debora Porri, Hellas Cena

**Affiliations:** ^1^Laboratory of Dietetics and Clinical Nutrition, Department of Public Health, Experimental and Forensic Medicine, University of Pavia, Pavia, Italy; ^2^Clinical Nutrition and Dietetics Service, Unit of Internal Medicine and Endocrinology, ICS Maugeri IRCCS, University of Pavia, Pavia, Italy

**Keywords:** pediatric obesity, eating disorders, multidisciplinary treatment, obesity prevention, obesity management

## Abstract

The prevalence of obesity in children/adolescents has increased worldwide during the past 30 years, becoming a significant public health concern; prevention, and management of pediatric obesity onset is one of the most critical public health goals for both industrialized and developing countries. Pediatric obesity has been identified as a risk factor for various psychopathologies, including eating disorders (ED). Although it has been demonstrated that a comprehensive multidisciplinary treatment (MT), with small steps and practical approaches to lifestyle change, can be an effective treatment for children and adolescents with obesity, to the best of our knowledge, this is the first systematic review investigating the effect of MT on the development, progression or decrease of ED symptoms (EDS) in this target population. PubMed and Web of Science databases were searched (last search on 18 February 2019) according to a predetermined search strategy, in accordance with the Preferred Reporting Items for Systematic Reviews and Meta-Analyses (PRISMA) Guidelines and Statement. Original studies published in English examining the effect of MT on pediatric overweight/obesity, paying particularly attention at the development of EDS, were eligible for inclusion. Seven hundred and forty-four records have been identified; nine articles with study quality ranging from weak to moderate have been included. MTs were heterogeneous in nature including length, number, frequency and type of sessions, parent-involvement and use of technology, besides several psychometric questionnaires were used to screen for EDS, since there are no standardized criteria. In 3 studies there was a significant decrease in external and emotional eating and in four studies a significant increase in restraint eating post MT. Two studies found a significant decrease of binge eating symptoms and other two studies showed an improvement of self-perception, weight, and shape concern. A statistical significant decrease in BMI, BMIz, BMISDS, and adjusted BMI was observed after all MTs, except one. A narrative summary of the evidences reported highlighted the positive impact of MT on the EDS. Moreover, since weight loss post MTs was not necessarily related to EDS, clinicians should also look for the presence of EDS and treat them accordingly.

## Introduction

Pediatric obesity is one of the greatest health challenges of the Twenty-first century (1). Prevalence of overweight or obesity in infants and young children (0–5 years) has increased globally and rapidly over time from 32 million in 1990 to 41 million in 2016 (2). In the United States, more than 10% of infants or toddlers and more than 17% of children and teens are affected by obesity while in Europe (3), 19–49 and 18–43% respectively of boys and girls are affected by either overweight or obesity (4). If the current rates keep on rising, the number of infants and young children with overweight or obesity will reach 70 million by 2025 (2). In addition, evidence show that at least 25–50% of children and adolescents defined as having a healthy Body Mass Index (BMI)-for-age have excessive body fat and this may indicate a dangerous underestimation of “obesity” comorbidities risk (5–7). Indeed, childhood and adolescent obesity is associated with a number of negative health sequelae, including hypertension, hyperlipidemia, respiratory problems, endocrine consequences, orthopedic complications, which contribute to a significant decrease in quality of life and life expectancy (8).

Recently, the Childhood Obesity Task Force (COTF) of the European Association for the Study of Obesity (EASO) classified obesity as not just a health risk but as a chronic disease in children and adolescents, in order to develop tailored interventions and health policies to prevent and treat obesity at both public and individual level (1).

Furthermore, there is increasing evidence that childhood-onset obesity is not only a risk factor for metabolic complications in adulthood, but also associated with an increased risk of developing eating disorders (EDs) during adolescence (9–13).

Children with obesity or overweight experience psychosocial distress that significantly affects their quality of life and well-being, so mental health of these children has also gained the attention of researchers and clinicians (11, 14–16).

Additionally, pediatric obesity has been identified as a risk factor for psychopathology, that may manifest itself through eating disorder symptoms (EDS) like excessive shape and weight concerns, body image dissatisfaction, dieting, and other unhealthy weight control methods, or binge eating (17, 18), sneaking, hiding or hoarding food, eating in the absence of hunger and inhibition or embarrassment when eating in front of others (19).

ED symptoms do not always correspond in severity or specificity to full-syndrome ED (20); they encompass a broad array of dimensional maladaptive cognitions and behaviors relating to eating and weight, that are found across the range of full syndrome ED diagnoses as well as in subsyndromal variants (21). This is probably why they are continuously underdiagnosed by pediatric professionals, although they are more common than metabolic disorders in childhood and adolescence and are associated with high morbidity and mortality (20). Overweight adolescents have a 2 1/2 to 5 times higher risk of developing eating disorders than teens whose weight is in the healthy range (21, 22).

Prevention and management of obesity onset is one of the most critical public health goals, and childhood represents the ideal time for lifestyle intervention, throughout multidisciplinary treatment (MT), as lifestyle habits in youth are not yet ingrained (23, 24). In fact, evidences show that behavioral lifestyle interventions are effective for weight loss in most children and adolescents (25). Success in treatment of childhood obesity requires a multifaceted approach to nutrition patterns and physical activity, with particular attention paid to the family and other environmental factors that may significantly affect outcomes (26).

The 2016 WHO Commission on Ending Childhood Obesity report, recommended “family-based, multicomponent, lifestyle weight management services for children and young people who are obese” as part of the universal child and adolescent health care (2). There is no specific definition of “multidisciplinary treatment” (MT), but some authoritative sources [WHO (2); Endocrine Society (27)] underlined it should include some common components such as nutrition and physical activity, besides family counseling and psychosocial support.

The multidisciplinary approach should also include health professionals such as physicians, dieticians, health coaches, and psychologists or other mental health care providers able to offer behavioral counseling (28, 29). Working in teams allows for modification of assessment and treatment providing effective integral interventions in the management of childhood obesity.

Multidisciplinary treatments (MTs) have proven beneficial and effective in combating obesity reducing body mass index (BMI) as well as the risk of future comorbidities (30–32).

Treatment of overweight and obesity mainly aims at achieving weight loss and BMI reduction, fat mass decrease, risk factors for metabolic syndrome decline and increased health-related quality of life (33, 34). However, despite weight reduction is a common and legitimate outcome to pursue, psychosocial contributors to eating behaviors not be neglected (35–37).

### Objectives

Since the effects of MT on eating behavior in children with overweight/obesity are largely unknown, in this systematic review, we attempted to address the question: is multidisciplinary treatment effective on eating disorder symptoms in children with obesity?

This systematic review has been conducted following the Preferred Reporting Items for Systematic Reviews and Meta-Analyses (PRISMA) guidelines recommending to present a full electronic search strategy for at least 1 major database (38).

## Methods

### Data and Search Strategy

Two electronic databases (PubMed and Web of Science) were searched from 2008 to 2019 using the following structured search strings: childhood obesity OR childhood overweight OR pediatric obesity OR pediatric overweight OR obesity in children OR overweight in children OR multidisciplinary treatment^*^ OR multidisciplinary approach^*^ OR multidisciplinary intervention^*^ OR multidisciplinary program^*^, combined to eating behavior^*^ OR binge eating OR sneaking food^*^ OR hiding food^*^OR hoarding food^*^ OR reward OR overeating.

### Eligibility Criteria

All studies were assessed according to the following inclusion and exclusion criteria summarized below:

#### Participants

Eligible participants were children with overweight/obesity (as defined in the selected studies), age ranging from 6 to 18 years at the beginning of MT. Participants with pre-existing disease or organic cause for obesity and on medications that could affect weight were excluded.

#### Intervention

MT was defined as an approach covering lifestyle intervention on nutrition and healthy behavior dietary patterns and/or physical activity. According to this, we selected only studies that included MTs considering also EDS.

Assessment of EDS was obtained through a variety of different psychometric tests: DEBQ (Dutch Eating Behavior Questionnaire) (39), ChEDE (Child Eating Disorder Examination) (40), YEDE-Q (Youth Eating Disorder Examination Questionnaire) (41, 42), CEBQ (Children Eating Behavior Questionnaire) (43), TEFQ (Three-Factor Eating Questionnaire) (44), BES (Binge Eating Scale) (45), BITE (Bulimic Investigatory Test, Edinburgh) (46), EDI—II (Eating Disorder Inventory) (47), EES-C (Emotional Eating Scale for Children and Adolescents) (48), QWEP (Questionnaire on Weight and Eating Patterns) (49), EI (Eating Inventory) (50).

#### Comparison

Different study designs (i.e., randomized controlled-trials, case–control studies and pre–post uncontrolled studies with no comparison group) were included in this review.

#### Outcome

The outcome of this systematic review was to evaluate for the first time MT impact on EDS in children affected by overweight/obesity.

We also evaluated weight reduction and/or fluctuations, expressed as Body Mass Index (BMI), BMI z score (BMIz), BMI Standard Deviation Score (BMISDS) as a possible confounding factor.

#### Exclusion Criteria

The comprehensive search strategy inadvertently retrieved studies that were unrelated to the aim of this systematic review and were subsequently excluded. Narrative reviews, systematic reviews and case reports were excluded, as well as case series, descriptive studies, letters, comments, articles that did not correspond to the objective of this review or had no full-text accessible in English.

### Selection Process

Titles and abstracts were screened by two authors (Di Napoli Ilaria, Porri Debora) for inclusion. Reference lists of primary articles and related reviews were checked to identify any other study appropriate for inclusion. Studies assessed as eligible, potentially eligible or unclear, were retrieved in full text whenever available. Any uncertainty concerning the inclusion of specific studies was resolved by discussing with a third author (De Giuseppe Rachele). Last search date 18/02/2019.

### Data Extraction

Study's characteristics (e.g., multidisciplinary treatment, participants, aim, outcome of interest, and study design) were extracted into standardized tables. Data items extracted were used to investigate the effect of MT on EDS in children affected by overweight/obesity.

### Data Synthesis

Due to the heterogeneity of study population characteristics and MTs features (such as length of the treatment, outcomes measured, and timing of assessment), we were not able to perform a meta-analysis. However, we conducted a narrative summary of the findings.

### Quality Assessment and Risk of Bias

Study quality was assessed in duplicate using a designed appraisal tool, the Effective Public Health Practice Project Quality Assessment Tool for Quantitative Studies, a useful tool for systematic reviews, which evaluates randomized and non-randomized intervention studies (51).

Individual component quality rankings, including the risk of bias measures are included in the Supplementary Table 1.

Component and overall quality ratings were scored as “strong,” “moderate” or “weak” according to instructions accompanying the tool (52, 53).

## Results

### Overview of Studies

**Figure 1:**
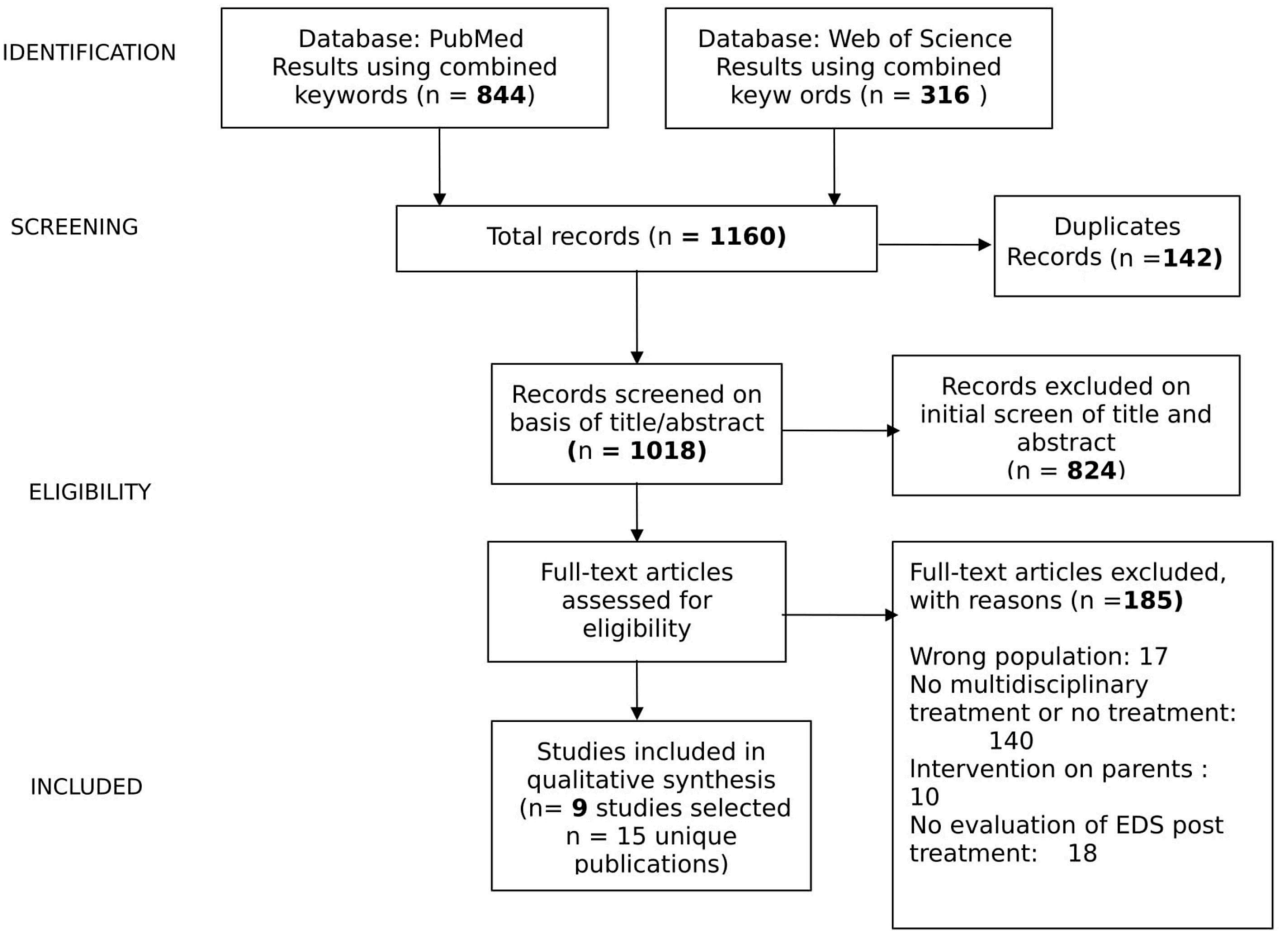
Studies selection.

A flowchart summarizing the study selection procedure is presented in Figure 1.

Electronic searches returned 1,160 records. Duplicates (*n* = 142) were firstly removed. Secondly, 194 studies were retained after screening the titles and abstract. Finally, 170 studies were further excluded after reading throughout full texts. Of the 184 excluded records, 17 referred on a wrong population, in 140 MT was not considered, in 10 was considered an intervention on parents and in 18 were not evaluated EDS post MT. Only 9 eligible studies met the inclusion criteria and were included in this review.

Data abstraction revealed 6 programs that had multiple publications either protocols, additional cohorts, further follow-up time-points or different outcome measures/other secondary analysis (see references in Supplementary Table 1). Hence, studies were grouped by program cohort for reporting and analysis. Disagreements were resolved via discussion.

### Study Characteristics

Characteristics of selected studies are reported in Table 1. All studies have been published between 2008 and 2019.

**Table 1:** Characteristics of selected studies.

**References**	**Study design**	**Quality score**	**Simple size**	**Age**	**Questionnaire**
Cohen et al. (54)	Randomized Controlled Trial	moderate	78	6–8 year old	CEBQ(43)
Balantekin et al. (55)	Interrupted time series without comparison group	moderate	241	7–11 years old	ChEDE (40) YEDE-Q (41, 42)
Halberstadt et al. (56)	Interrupted time series without comparison group	weak	120	8–19 years old	DEBQ (39)
Adam et al. (57)	Interrupted time series without comparison group	weak	604	10 – 15 years old	TFEQ (44)
Raimunda Damaso et al. (58)	Interrupted time series without comparison group	moderate	97	15−19 years old.	BES (45) BITE (46)
De Niet et al. (59)	Randomized Controlled Trial	moderate	144	8–12 years old.	DEBQ (39)
Bishop-Gilyard et al. (60)	Randomized double blinded placebo–controlled trial	moderate	82	13–17 years old	QWEP (49) EI (50)
Goossens et al. (61)	Interrupted time series without comparison group	weak	108	10–17 years old	ChEDE (40)
Sarvestani et al. (62)	Non Randomized Controlled Trial	moderate	60	11-15 years old.	DEBQ (39)

Four studies were conducted in Europe (56, 57, 59, 61), two studies took place in US (55, 60) while one study was conducted in Canada (54), one study was conducted in Brazil (58), and one in Iran (62).

The programs were evaluated as controlled trials (*n* = 4) (54, 59, 60, 62) both randomized (*n* = 3) (54, 59, 60) and non-randomized (*n* = 1) (62), and as interrupted time series without comparison group (*n* = 5) (55–58, 61).

Study quality was assessed to be weak for three studies (56, 57, 61); moderate for six (54, 55, 58–60, 62). Eight studies (54–57, 59–62) were rated as being weak for selection bias as they involved self-referrals from community advertisements and public service announcements, as is commonplace for community interventions, and thus participants were not randomly selected from the eligible population.

The sample size ranged from 60 to 504 children and adolescents while age of participants ranged from 7 to 18 years old.

In all studies, selected multidisciplinary treatment of pediatric obesity was provided. MTs were heterogeneous in nature, including length, number, frequency and type of sessions, parent-involvement and technology involvement.

Among selected studies, eight of them (54–57, 59–62) required either active and frequent participation of at least one parent/caregiver during MT or a “family-based intervention.”

As regards the characteristics of the various MT approaches, all treatments (54–62) included either advice on healthy nutritional habits or advice on physical activity and healthy lifestyle.

Particularly, in one study (54), besides nutritional and lifestyle advice, both dietary treatment and structured physical activity were performed, while in other three studies (58, 61, 62) besides lifestyle advice, MT included the prescription of structured physical activity sessions, under the supervision of a skilled trainer.

Additionally, four studies (56, 57, 60, 61) combined an inpatient multidisciplinary program, ranging from 6 weeks to 10 months, with a strict control of children's daily dietary intake and lifestyle habits. De Niet et al. (59), evaluated the effect of Short Message Service (SMS) maintenance treatment (SMSMT) by means of smart devices.

Finally, only one study reported the use of medications (e.g., sibutramine) during MT (60).

Concerning psychological aspects in MT, all studies selected in this review (54–62) included support group therapy, with exception of Adam's (57) study for which we were not able to establish it since the protocol, described previously elsewhere, was written in German. Only Raimunda Damaso et al. (58) included also individual therapy in MT.

All MT approaches (54–62) included cognitive behavioral therapy (CBT), with parents' involvement promoting parent-child interaction patterns change toward a supporting relationship, instead of a parental controlling behavior, giving positive feedback and reinforcement. Three (56, 59, 61) of these nine studies analyzed also the psychosocial consequences of obesity and the relationships of children with their peers. Considering the duration of the various MT approaches, except one study (55) that did not state MT length, we reported different lengths of treatments ranging from 16 weeks (62) to 12 months (54, 56–60). As for follow ups three studies (56, 57, 61) reported follow ups ranging from 12 to 60 months post MT.

### Outcome

The focus of this review was to evaluate MT outcome on ED symptoms in children affected by overweight/obesity (**Table 4**).

Different questionnaires have been used (Table 2) and administered at baseline, at the end of MT and during follow-up for ED symptoms screening in children and adolescents, since the lack of a standardized method.

**Table 2:** Description of questionnaires used in the different selected studies to assess ED symptoms.

**References**	**Variables assessed**	**Questionnaires**
Cohen et al. (54)	Items aimed at investigating: Food Approach: food responsiveness, enjoyment of food, emotional overeating and desire to drink Food Avoidance: headings slowness in eating, food fussiness, satiety responsiveness and emotional under eating.	CEBQ (43) Children Eating Behaviour Questionnaire
Balantekin et al. (55) Goossens et al. (61)	Items aimed at investigating four major areas of eating disorder psychopathology: restraint, eating, shape, and weight concerns.	ChEDE (40) Child Eating Disorder Examination
Balantekin et al. (55)	Adaption of ChEDE for adolescents.	YEDEQ (41, 42) Youth Eating Disorder Examination Questionnaire
Halberstadt et al. (56) De Niet et al. (59) Sarvestani et al. (62)	Items aimed at investigating: external eating, emotional eating and restrained eating.	DEBQ (39) Dutch Eating Behaviour Questionnaire
Adam et al. (57)	Items aimed at evaluating: disinhibition, cognitive control, flexible control and rigid control.	TFEQ(44) Three-Factor Eating Questionnaire
Raimunda Dâmaso et al. (58)	Items aimed at evaluating Bulimia symptoms.	BITE (46) Bulimic Investigatory Test, Edinburgh
Raimunda Damask et al. (58)	Items aimed at describing both behavioral manifestations and feeling/cognitions surrounding a binge episode and cognitive phenomena thought to be related to binge eating.	BES (45) Binge Eating Scale
Bishop-Gillard et al. (60)	Items aimed at measuring the ability to control food intake, loss of control over eating, and reported hunger	EI (50) Eating Inventory
Bishop-Gillard et al. (60)	Items aimed at measuring the BED diagnostic criteria.	QWEP(49) Questionnaire on Weight and Eating Patterns
Goossens et al. (61)	Items aimed at assessing both eating attitudes and related ego dysfunction characteristics. For the purpose of this study, nag eating attitudes (i.e., drive for thinness, bulimia, and body dissatisfaction) were evaluated	EDI-II (47) Eating Disorder Inventory

MT features as sessions, length, follow-up, focus, prescriptions, target, and parental involvement are described in Table 3 while the outcome of selected studies is reported in Table 4.

**Table 3:** Description of studies' multidisciplinary treatments.

**References**	**Multidisciplinary treatment**	**Sessions**	**Length**	**Follow-up**	**Focus**	**Diet or physical activity prescribed by a specialist**	**Inpatient period**	**Parents involved**
Cohen et al. (54)	Based on Canadian diet and physical activity guidelines. Children were randomized into 3 groups: – *Control* (Ctrl; no intervention) – *Standard treatment* (StnTx: 2 servings milk and alternatives/day (d), 3x/week. weight bearing physical activity) – *Modified treatment* (ModTx: 4 servings milk and alternatives/day; daily weight bearing physical activity). Ctrl received counseling after 12 months	StnTx and ModTx participated in 6 sessions, which were held at the end of each month for the first 5-months of the study, then a final “relapse prevention” session at the end of the 8th month. Ctrl group received the interventions after the end of the study.	12 months	NO	– Physical activity – Nutrition – Eating behavior – Parenting skills	YES (diet, structured physical activity)	NO	YES
Balantekin et al. (55)	Family-based behavioral weight loss treatment	16 session of family-based behavioral treatment.	Not specified	NO	–Nutrition, – Physical activity, – Eating behavior – Parenting skills	NO	NO	YES
Halberstadt et al. (56)	Combined multidisciplinary lifestyle intervention. Two months or 6 months period of inpatient treatment during weekdays requiring active and frequent participation of the parents/caregivers.	The MT had a period of inpatient treatment during weekdays of either 2 months and biweekly return visits of 2 days during the next 4 months or 6 months, followed by 6 monthly return visits of 2 days	12 months	12 months	– Nutrition, – Physical activity – Eating behavior – Parenting skills	NO	YES	YES
Adam et al. (57)	The DAK program, designed for one year with an initial multidisciplinary inpatient treatment followed by an outpatient family based treatment.	The details of MT was previously published elsewhere. The protocol was written in Germany (see Supplementary Table 1)	12 months	48 months	–Nutrition, – Physical activity – Eating behavior – Parenting skills	YES (diet and structured physical activity)	YES	YES
Raimunda Damaso et al. (58)	Multidisciplinary treatment with the supervision of an exercise physiologist	Once a week, the adolescents had classes on topics related to improved food consumption. Adolescents underwent therapy support group weekly Adolescents were involved in structured session of physical activity three times a week.	12 months	NO	– Physical activity – Nutrition – Eating behavior	YES (structured physical activity)	NO	NO
De Niet et al. (59)	SMS maintenance treatment (SMSMT) program After the first 3 months of treatment where children and parents were involved into educational session group, participants were randomly assigned to: – Intervention group, receiving SMSMT for 9 months, or to – Control group (no SMSMT)	1 intake session; 8 children sessions; 3 parent sessions; for 3 months.	12 months	NO	– Physical activity – Nutrition – Eating behavior – Technology involvement – Parenting skills	NO	NO	YES
Bishop-Gilyard et al. (60)	Participants attending at a family based behavioral weight loss program were randomly assigned to: –Intervention group (sibutramine 15 mg/d) or to – Control group received placebo.	The treatment was structured into 2 phases.Phase 1: Both intervention and control group attended a behavioral counseling for 4 months followed by bi-weekly visits for an additional 2 months. Parents were instructed in methods of supporting their children. Phase 2: After the initial 6 months all participant received sibutramine for 6 months.	12 months	NO	– Physical activity – Nutrition – Eating behavior – Parenting skills	YES (diet)	NO	YES
Goossens et al. (61)	Inpatient non-diet healthy lifestyle program.	Each child received 4 hours of individual guided exercises. All children had facilities to take part in exercise programs for at least 14 hours per week. All children received a 12-week cognitive behavioral treatment.	10 months	60 moths	– Physical activity – Nutrition – Eating behavior – Parenting skills	YES (structured physical activity)	YES	YES
Sarvestani et al. (62)	Participants were randomized into: – Intervention group receiving lifestyle counseling and structured sessions of physical activity – Control group attended only three sessions of the same treatment.	Four-hour structured sessions of physical activity were held weekly for 16 weeks; each session involved 2 hours of behavior modification or dietary instruction and 2 hours of yoga therapy.	4 months	NO	– Physical activity – Nutrition – Eating behavior – Parenting skills	YES (structured physical activity)	NO	YES

**Table 4:** Outcome of selected studies.

**References**	**Outcome**
Cohen et al. (54)	StnTx: - Food Approach ↓^**^ - Food Avoidance not significantly change - BMIz ↓^*^ ModTx: - Food Approach ↓ - Food Avoidance not significantly change - BMIz ↓ Ctrl: - Food Approach ↔ - Food Avoidance not significantly change - BMIz ↔^*^
Balantekin et al. (55)	Entire sample: - Weight concern ↓^**^ - Shape concern no significant change - LOC no significant change - BMIz↓^***^ HIGH and SWC (compared with LOW): - Weight concern ↓^***^ - Shape concern ↓^***^
Halberstadt et al. (56)	Girls: - Restraint eating ↑^**^ - External eating↓ Boys: - Emotional eating ↓^*^ - External eating ↓^**^ Entire sample: BMISDS ↓^***^
Adam et al. (57)	- Cognitive control/Restrained eating ↑^***^ - Flexible control ↑^***^ - Disinhibition ↓^***^ - Rigid control ↓(at 24 months and at 48 months)^***^ - BMISDS ↓^***^
Raimunda Damaso et al. (58)	- Percentage of adolescents with binge eating symptoms ↓^***^ - BMI ↓^***^
De Niet et al. (59)	- Emotional eating ↓^*^ - External eating ↓^*^ - BMISDS ↓^***^
Bishop-Gilyard et al. (60)	- Percentage of adolescents with binge eating symptoms ↓^**^ - Hunger ↓^***^ - Disinhibition ↓^***^ - Cognitive restraint ↑^***^ - BMI ↓
Goossens et al. (61)	- OBE ↔ - Restraint ↔ - Weight concern ↔ - Shape concern ↔ - SBE ↓ - Eating Concern ↓^**^ - Drive for Thinness ↓^**^ - Bulimia ↓^**^ - Body Dissatisfaction ↓^**^ - adjusted BMI ↓^***^
Sarvestani et al. (62)	- Emotional eating ↓^*^ - Restraint eating ↑^*^ - External eating ↓^*^ - BMI ↓^*^

SBE, subjective binge eating episodes; OBE, objective binge eating episodes; LOC, loss of control; HIGH, high probability to develop ED pathology group; SWC, shape and weight concern group; LOW, Low probability to develop ED pathology group; BMIz, BMI z score; BMISDS, BMI Standard Deviation Score; ↓ decrease; ↔ remain stable; ↑ increased.

* p ≤ 0.05;

** p ≤ 0.01;

*** *p ≤ 0.001*.

Additionally, we evaluated BMI fluctuations, expressed as BMI, BMIz, BMISD, or adjusted BMI, as a possible confounding factor, at end of treatment (MT) (54–62) and during follow-up period (56–58, 61) (Table 4).

Raimunda Damaso et al. (58) identified symptoms of binge eating and bulimia at baseline in 6% of their sample of adolescents with obesity, by means of BES and BITE questionnaires; at 12 months' follow up, the percentage of adolescents with binge eating symptoms had significantly decreased (2%) (Table 4).

Similarly, Bishop-Gilyard et al. (60) assessed symptoms of binge eating in adolescents affected by obesity by QWEP and EI questionnaires (Table 2), complemented by an interview to estimate the amount of food consumed during binging episodes and evaluate loss of control. The Authors (60) reported at baseline binge eating symptoms in 24% of participants with a significant decrease after 6 and 12-months' post MT, respectively 8 and 3%. Moreover, hunger and disinhibition significantly dropped, while cognitive restraint significantly increased over time (Table 4).

Concerning BMI reduction/fluctuation, the authors (60) also noticed that adolescents with obesity and binge eating episodes lost the same amount of weight as those without these episodes.

Besides binge eating, other eating disordered symptoms were identified by means of several others questionnaires as described in Table 2.

Choen et al. (54) conducted an RCT aimed at examining changes in EDS by means of CEBQ (Table 2) in children with overweight and obesity participating in a 12 months-MT protocol, based on a family-centered lifestyle intervention, according to Canadian dietary and physical activity guidelines. As shown in Table 3, the subjects were divided into three groups: control (Ctrl), standard (StnTx) and modified (ModTx) treatment. The StnTx and the ModTx attended 6 sessions plus a final of “prevention of relapse” session at the end the eighth month MT (Table 3) while the Ctrl group received the same session but at the end of 12 months of the study. CEBQ scores were then categorized as either Food Approach or Food Avoidance (Table 2), meaning for Food Approach food responsiveness, enjoyment of food, emotional overeating, and desire to drink scoring and for Food Avoidance slowness in eating, food fussiness, satiety responsiveness, and emotional under-eating scoring. Food Approach resulted significantly decreased only in the StnTx group and not in the ModTx group when compared to Ctrl group. Food Avoidance did not significantly change among groups (Table 4). Notably, this is the only study that used a parent-completed questionnaire (CEBQ).

Adam et al. (57) evaluated four parameters of EDS (disinhibition, cognitive control/restrained eating, flexible control, rigid control), by means of TFEQ questionnaire (Table 2) at baseline, at the end of MT and 24 and 48 months post treatment (MT). The Authors reported that cognitive control/restrained eating, flexible control, and disinhibition improved significantly at the end of MT as well as 24 and 48 months post MT, when compared to the baseline; rigid control improved but reached the significance only 24 and 48 months post MT (Table 4).

Goossens et al. (61) and Balantekin et al. (55) assessed different aspects such as restraint, eating, shape, weight concerns and loss of control (LC) overeating identified as objective binge eating (OBE) and subjective binge eating (SBE) on adolescents and children with overweight/obesity by using ChEDE, as described in Table 2. Balantekin et al. (55) used YEDE-Q (a version of ChEDE adapted for adolescent population) and EES-C to rate children desire to eat facing emotions; Goossens et al. (61) additionally used EDI-II (Table 2) aimed at assessing both eating attitudes and related ego dysfunction characteristics (such as drive for thinness, bulimia, and body dissatisfaction).

Previous findings (63) hypothesized that EDS in youngsters with overweight, would remain stable or decrease over a certain period post MT. Similarly, Goossens et al. (61) investigated the stability of EDS in youngsters at 60 months post MT, reporting that some ED symptoms (like OBE, Restraint, Weight, and Shape Concerns) remained stable, while others decreased (like SBE, Eating Concern, Drive for Thinness, Bulimia, and Body Dissatisfaction) (Table 4).

Balantekin et al. (55) quantified each different aspect (restraint, eating, weight and shape concerns and loss control) investigated through ChEDE questionnaire (Table 2), identifying at baseline 4 different EDS patterns: (i) LOW (subjects with a very low probability to develop ED); (ii) SWC (subjects with a high probability to develop shape and weight concerns); (iii) OLOC (subjects at risk of loss of control eating); (iv) HIGH (subjects with a high probability to develop ED). The Authors (55) reported that after 16 MT sessions (Table 4), there was a significant decrease in weight concern from baseline to post treatment for the entire sample, with a significant time-by-group interaction. Compared with LOW children, HIGH, and SWC ones reported significantly greater reductions in weight concern; no differences were detected between children in LOW and OLOC and no significant change in shape concern from baseline to post treatment was observed for the entire sample. However, there was a significant time-by-group interaction; in fact, compared to LOW children, the HIGH, and SWC ones experienced a significantly greater reduction in shape concern. No significant change in the number of LOC eating episodes nor in the time-by-group interaction was found for the entire sample (Table 4). Interestingly, although the significant reduction in BMIz and weight concern after the MT in the whole study sample, Balantekin et al. (55) reported a lower decrease in BMIz in HIGH and SWC group when compared to LOW group. Moreover, shape and weight concern in HIGH group was not significantly related to BMIz reduction (55).

Finally, three studies (56, 59, 62) used DEBQ questionnaire (Table 2), in order to evaluate external, emotional and restrained eating in children and/or adolescent with overweight/obesity post MT.

Particularly, a RCT performed by de Niet et al. (59) and a non-RCT performed by Sarvestani et al. (62) reported a significant reduction in the emotional eating after 12 months and 6 months' MT, respectively (Table 4). Sarvestani et al. (62) also observed a significant increase in restraint eating (Table 4).

Similarly, Halberstadt et al. (56) in an interrupted time series with no control group study, noticed a significant increase of restraint eating in girls and, in agreement with others (62), a significant decrease in emotional eating but only in boys (Table 4).

The authors (56) also reported a significant reduction of BMI and BMISDS post MT that was maintained during follow-up. However, a slight increase in BMI and BMISDS occurred after 12 months, showing that weight re-gain influenced significantly the increase of restraint and external eating post MT, only in girls.

Concerning external eating, all studies (56, 59, 62) reported a significant decrease after MT (Table 4).

## Discussion

Multidisciplinary treatment for children/adolescents with overweight and obesity should focus on diet and healthy eating habits, physical activity, and family coping strategies (64, 65).

Assessment and treatment of childhood obesity and associated medical conditions, including psychological consequences, should be ensured.

It is well known that psychological distress and risk for eating disorders in pediatric population with a history of obesity are frequent (19); however classifying eating disorders in youth is challenging (66).

During childhood, eating disorders often present with atypical or sub-threshold criteria. This is particularly true for Binge Eating, one of the most common ED symptom associated with childhood obesity (19). Despite binge eating symptoms in children with obesity are common, the diagnosis of binge eating disorder (BED) in youth is rare (67–69); compulsive eating and stereotypical disordered eating behaviors such as hiding food, eating in secret, purging, exercising excessively, can be frequently observed (20).

Previous research showed an association between pediatric obesity and EDS, such as dietary restraint, self-dieting, and body image dissatisfaction (70). Moreover, results confirmed by Boutelle et al. (71) suggested that external eating, satiety sensitivity, eating in the absence of hunger, loss of control eating, and emotional eating were related to adiposity and excessive weight gain in children.

Assessment and management of EDS caused by or consequent to excessive weight gain are not always contemplated with due caution; however, pediatric obesity should be considered a significant risk factor for the development of eating disorders during adolescence and childhood (17).

Although multidisciplinary treatments are well supported by the literature in their effectiveness to reduce BMI and risk of future co-morbidities (30–32), to the best of our knowledge the effect of MT on the development, progression or reduction of EDS in children/adolescents with overweight/obesity has never been evaluated.

In our systematic review, the first aim was to evaluate the effectiveness of MT on EDS in children with obesity.

Concerning MT efficacy on dietary restraint, results (56, 57, 60, 62) presented are conflict.

Adam et al. (57) found a significant decrease of dietary restraint after MT; they also reported a significant reduction of flexible control, a typical behavior characterized by a graduated “more or less” approach to eating and weight control, which is considered a permanent behavior (57).

These effects were also maintained during the whole follow-up period (57) and can reflect a success of MT since dietary restraint is often considered a determinant of overeating and a precursor of EDS (72). In fact, as previously demonstrated by Stice et al. (73) in a 60 months prospective study on 496 adolescent girls, children and adolescents with higher dietary restraint scores appeared to have an increased risk of developing obesity later on.

On the contrary, other findings (56, 60, 62) described in our systematic review, reported that children or adolescents with obesity, especially girls (56) showed a significant increase in dietary restraint after MT.

However, in the light of what has been described above, it is also important to note that while dietary restraint is often conceptualized as maladaptive for individuals with ED, in the context of obesity, a moderate degree of dietary restraint may be beneficial in facilitating weight loss, improving physical health and maintaining weight control after treatment (74).

It should be noted that dietary restraint scales measure the intention to eat less rather than the real energy intake restriction (75); although some people may develop an intention to restrict their food intake, this intention is not always translated into action (73, 75). Planning, maintenance self-efficacy and action control are suggested to be important variables that may explain the gap between intention and behavior; subjects showing higher food restriction intention are more vulnerable to future eating disorders and weight gain (76).

Similarly, self-dieting is common among adolescents but it is not always a harmless behavior (17, 77). In fact, if self-dieting is not supervised or controlled, may lead to negative emotions that increase the risk of binge eating and use of inappropriate compensatory behaviors (17, 78).

Additionally, binge eating is a cognitive and behavioral process that is particularly important in the context of obesity (74), as recently demonstrated by a meta-analysis (79) binge eating symptoms are prevalent in more than one quarter of children and adolescents with overweight and obesity (19, 79).

Goossens and Bishop-Gilyard (60, 61) found a significant decrease in binge eating symptoms in adolescents post MT, confirming the importance of a structured MT rather than self-made -diets. They also distinguished between OBE, with the onset of LC over eating a larger amount of food that other people would not do, and SBE, with the onset of LC over eating a subjective large amount of food, that other people would not quantify as unambiguously large (61). The authors (61) reported a decrease of SBE after MT while OBE did not change (Table 4). This may occur since SBE seems quite common in youngsters, and its association with obesity and psychological impairment has already been demonstrated (80) while OBE is more common in adults (80).

Moreover, children and adolescents with obesity can develop body dissatisfaction which is linked to unhealthy weight control behaviors, binge eating, and lower physical activity levels (81); in addition, children and adolescents with obesity or overweight may experience weight stigma that exacerbates weight gain and creates additional barriers to healthy behavioral changes (82).

However, concern for body image, which is central to adolescents' overall sense of self-esteem, can play a dual role according to personal and environmental interactions (83, 84). In fact, it may exert negative or positive feedback, respectively, pushing toward dieting and triggering overeating or acting as a motivational driver toward healthier eating and lifestyle behaviors (83, 84). Studies reported in our systematic review (55, 61) assessed concern for body image throughout different questionnaires, investigating self-perceptions, weight, and shape concern. Only Balantekin et al. (55) and Goossens et al. (61) showed an improvement of self-perception, weight and shape concern, post MT.

Among the EDS, emotional and external eating can be considered behaviors related to overeating. Emotional eating means eating in response to emotional states, such as hunger, fear or anxiety, while external eating identifies eating in response to environmental food stimuli, such as sight and smell of food, regardless of hunger, and satiety stimuli (85).

Moreover, disinhibition leads to increase food intake and overeating if exposed to emotional stimuli (57).

Studies described in our systematic review (56, 57, 59, 62) showed that MT had a positive impact on external eating, disinhibition of control and emotional eating. Many authors (56, 57, 59, 62) concluded by agreeing that MT could influence eating behavior and that children/adolescents with overweight/obesity undergoing MT learned to react to emotional stress and external stimuli.

In this systematic review, we also investigated the weight and/or BMI reduction/fluctuation, as possible confounding factor.

A statistically significant decrease in BMI was observed in all MT approaches (54–62) except for Bishop-Gilyard et al. (60) where the decrease did not reach the significance.

Some authors (55, 56, 60) also analyzed the relation between EDS, such as weight and shape concern (55), binge eating episodes (60), dietary restraint, and external eating (56) and BMI reduction and/or fluctuation post MT.

Balantekin et al. (55) reported that BMIz was not strictly related to weight and shape concern improvement. In fact, although the authors (55) described a significant reduction in BMIz and weight concern post MT in the whole study sample. They reported a lower reduction in BMIz in subjects with a high probability to develop ED and in subjects with a greater likelihood of developing weight and shape concerns, when compared to subjects with a low risk of ED. Moreover, shape and weight concern in subjects with at high risk of ED was not significantly related to BMIz reduction (55).

Bishop-Gilyard et al. (60) revealed that adolescents with obesity and binge eating episodes lost the same amount of weight as those without binge.

Halberstadt et al. (56) described a BMI re-gain impact on EDS, showing that weight re-gain affected the increase of restraint and external eating post MT, only in girls.

Therefore, given the small numbers of the studies mentioned above (55, 56, 60), we could only hypothesize that weight status and EDS improvement after MT were independent factors.

Notably, all selected studies (54–62) provided MT approaches including CBT and eight of them (54–61) considered either active and frequent participation of the parents/caregivers during MT or “family-centered” approaches.

Multiple organizations, including the American Dietetic Association, the American Academy of Pediatricians, and the National Academy of Medicine, support family-based treatments, encouraging healthy nutrition and parent education/modeling (86, 87). Nowadays, supporting parents to improve their skills regarding healthy child growth is considered an important public health goal (65, 88). In fact, it is well known (89) that parents impact child's behaviors throughout home environmental influence. For instance, parenting practices and lifestyle such as eating pattern, provision of nutritious food, physical activity reinforcement, and counteracting sedentary behaviors could influence children eating behavior. On the other hand, family-based interventions are an effective method, positively impacting pediatric obesity treatment outcome and improving the probability for children and adolescents to be adherent and successful once engaged in a weight loss program (90, 91).

### Strength and Limitations

The strength of this systematic review includes the development of a comprehensive search strategy applied, for the first time, on the effect of MT on the development and/or progress of EDS in children with overweight/obesity.

The assessment of risk of BIAS reported that the quality of the included studies was variable. Weak studies were included in this review and study quality was generally limited by participants selection (see Table S1, column “selection bias”). In fact, participants were no more likely to be representative of the target population, according to the tool that was used in this systematic review (51–53); in the selected studies, participants are often referred from a clinic or self-recruited via fliers, newspapers, television, radio, referrals from schools and community providers.

Another limit is that MTs were largely heterogeneous in length, period of discharge of patient, frequency and intensity of sessions, parents' involvement. Finally, quite a number of behavioral and psychosocial variables were assessed, by means of different validated questionnaires.

## Conclusions

Evidence showed that comprehensive MTs for children and adolescents with obesity reduce BMI and risk of future co-morbidities.

Although obesity and ED have traditionally been conceptualized as separate conditions, EDS in the pediatric population, with a history of obesity, are not unusual. Overweight and obesity in childhood and adolescence significantly increase the risk for ED development.

Results from this systematic review highlighted, for the first time, the positive short- and long-term impact of MTs on ED symptoms, which are not always associated to BMI reduction in children. Therefore, awareness amongst clinicians who treat children and adolescents with overweight and obesity should be raised so that EDS could be identified and treated accordingly.

## Author Contributions

RD, ID, and DP designed the search strategy. ID and DP screened studies for inclusion. RD resolved any uncertainty concerning the inclusion of specific studies. All authors analyzed results and drafted the manuscript. HC approved the manuscript. All authors are in agreement with the manuscript and declare that the content has not been published elsewhere.

### Conflict of Interest Statement

The authors declare that the research was conducted in the absence of any commercial or financial relationships that could be construed as a potential conflict of interest.
