# Supplementary material for: Pediatric Obesity and Eating Disorders Symptoms: The Role of the Multidisciplinary Treatment. A Systematic Review
Source: Front Pediatr. 2019 Apr 3;7:123. doi: 10.3389/fped.2019.00123 (PMC6463004; doi:10.3389/fped.2019.00123)
Supplement: Supplementary file 1 [file Table_1.docx]

# Supplementary Table 1: Study quality of selected studies, assessed by using Effective Public Health Practice Project Quality Assessment Tool for Quantitative Studies^(52-54)^.

| **Reference** | **Component Ratings** | | | | | | **Global Rating** |
| --- | --- | --- | --- | --- | --- | --- | --- |
|  | **Selection Bias** | **Study**  **Design** | **Confounders** | **Blinding** | **Data Collection**  **Method** | **Withdrawals**  **& Dropouts** |  |
| Cohen *et al.* 2013**^a^**  Choen *et al.* 2018^(61)^ | Weak | Strong | Strong | Strong | Strong | Strong | Moderate |
| Hayes *et al.* 2016**^b^**  Balantekin *et al.* 2017^(59)^ | Weak | Moderate | Strong | Moderate | Strong | Moderate | Moderate |
| Halberstadt *et al.* 2013**^c^**  Halberstadt *et al.* 2016^(58)^ | Weak | Moderate | Strong | Weak | Strong | Moderate | Weak |
| Adam *et al.* 2013^(55)^ | Weak | Moderate | Weak | Moderate | Strong | Weak | Weak |
| Raimunda Damaso *et al.*2013^(62)^ | Strong | Moderate | Weak | Moderate | Strong | Moderate | Moderate |
| Van den Akker *et al.* 2007**^d^**  De Niet *et al*. 2012^(56)^ | Weak | Strong | Strong | Moderate | Strong | Moderate | Moderate |
| Berkoviz *et al.* 2003**^e^**  Bishop-Gilyard *et al.* 2011^(60)^ | Weak | Strong | Strong | Strong | Strong | Strong | Moderate |
| Braet *et al.* 2004**^f^**  Goossens *et al.* 2011^(57)^ | Weak | Moderate | Strong | Moderate | Strong | Weak | Weak |
| Sarvestani *et al.* 2009^(63)^ | Weak | Strong | Strong | Moderate | Strong | Strong | Moderate |

**Legend.**

N/A, not applicable; Strong = no weak ratings; Moderate = 1 weak rating; Weak = ≥2 weak ratings

**Protocols.**

1. Cohen TR, Hazell TJ, Vanstone CA, Plourde H, Rodd CJ, Weiler HA. A family-centered lifestyle intervention to improve body composition and bone mass in overweight and obese children 6 through 8 years: a randomized controlled trial study protocol. BMC Public Health (2013) 13:383.
2. Hayes JF, Altman M, Kolko RP, Balantekin KN, Holland JC, Stein RI et al. Decreasing Food Fussiness in Children with Obesity Leads to Greater Weight Loss in Family-Based Treatment. Obesity (2016) 24:2158–2163. doi:10.1002/oby.21622.
3. Halberstadt J, Makkes S, de Vet E, Jansen A, Nederkoorn C, van der Baan-Slootweg OH et al. The role of self-regulating abilities in long-term weight loss in severely obese children and adolescents undergoing intensive combined lifestyle interventions (HELIOS); rationale, design and methods. BMC Pediatrics (2013) 13:41.
4. Braet C, Tanghe A, Decaluwé V, Moens E, Rosseel Y. Inpatient Treatment for Children With Obesity: Weight Loss, Psychological Well-being, and Eating Behavior. Journal of Pediatric Psychology (2004) 29(7):519-529.
5. van den Akker ELT, Puiman PJ, Groen M, Timman R, Jongejan MTM, Trijsburg W. A Cognitive Behavioral Therapy Program for Overweight Children. J Pediatr (2007) 151:280-3.
6. Berkowitz RI, Wadden TA, Tershakovec AM, Cronquist JL. Behavior therapy and sibutramine for the treatment of adolescent obesity: a randomized controlled trial. JAMA (2003) 289:1805-1812.
